# Development of a Blood Pressure Measurement Instrument with Active Cuff Pressure Control Schemes

**DOI:** 10.1155/2017/9128745

**Published:** 2017-10-03

**Authors:** Chung-Hsien Kuo, Chun-Ju Wu, Hung-Chyun Chou, Guan-Ting Chen, Yu-Cheng Kuo

**Affiliations:** ^1^Department of Electrical Engineering, National Taiwan University of Science and Technology, Taipei, Taiwan; ^2^Department of Biomedical Engineering, National Defense Medical Center, Taipei, Taiwan

## Abstract

This paper presents an oscillometric blood pressure (BP) measurement approach based on the active control schemes of cuff pressure. Compared with conventional electronic BP instruments, the novelty of the proposed BP measurement approach is to utilize a variable volume chamber which actively and stably alters the cuff pressure during inflating or deflating cycles. The variable volume chamber is operated with a closed-loop pressure control scheme, and it is activated by controlling the piston position of a single-acting cylinder driven by a screw motor. Therefore, the variable volume chamber could significantly eliminate the air turbulence disturbance during the air injection stage when compared to an air pump mechanism. Furthermore, the proposed active BP measurement approach is capable of measuring BP characteristics, including systolic blood pressure (SBP) and diastolic blood pressure (DBP), during the inflating cycle. Two modes of air injection measurement (AIM) and accurate dual-way measurement (ADM) were proposed. According to the healthy subject experiment results, AIM reduced 34.21% and ADM reduced 15.78% of the measurement time when compared to a commercial BP monitor. Furthermore, the ADM performed much consistently (i.e., less standard deviation) in the measurements when compared to a commercial BP monitor.

## 1. Introduction

With the rapid development of the economy, people's eating and living habits are changing. Less exercise and high-calorie foods gradually threaten human health [1]. In recent years, many reports and studies indicated that the ages of chronic patients are significantly reduced. Hypertensions are the precursors of many chronic diseases, such as strokes, heart diseases, kidney diseases, and retinopathies. Therefore, regular measurement of blood pressure has become one of the most important references of health.

Blood pressure measurement approaches have been widely discussed. Nitzan et al. [2] proposed a cuff-based automatic measurement of SBP which was based on the simultaneous measurement of photoplethysmography (PPG) signals [3] on the fingers of both hands. The PPG-based measurement directly detected the opening of the arteries under the cuff and provided accurate measurements of SBP. Song et al. [4] proposed a new cuff unit which improved the phenomenon of venous congestion for the measurement of the finger artery, and it was suitable for noninvasive and long-term monitoring. Due to the convenience of measuring finger arteries [5, 6], a lot of relative researches were proposed. However, the accuracy could be a concern. Lee et al. [7] proposed a calibrated method [8] which decreased the errors of different circumferences of the fingers of the subjects.

Moreover, Van Moer et al. [9] proposed a simplified method to obtain SBP and DBP. Based on the oscillometric method, blood pressure waveforms were analyzed in the frequency domain to filter out the harmonics and intermodulation products. In their work, the accurate estimations of SBP and DBP were obtained from oscillometric waveforms. Wang et al. [10] presented a model-based fuzzy logic controller for continuously noninvasive BP waveform monitoring. The model-based fuzzy logic controller was applied to track unknown desired trajectories and to find the optimal coupling condition where the maximum compliance of the arterial vessel occurred.

The oscillometric method is the most common approach [11, 12] to be applied to electrical BP measurement instruments. It has the advantages of noninvasive measurement which requires less professional skills. Therefore, the oscillometric method is suitable for daily health monitoring [13, 14] and long-term measurements [15, 16]. Practically, the user may place a sphygmomanometer cuff around the upper arm. The oscillations caused by heart pumping are changed with the altered pressure in the cuff. The observed pressure oscillations are measured with an electronic pressure sensor. When the cuff pressure is close to the mean blood pressure (MBP), the peak of oscillometric curve would occur.

**Figure 1:**
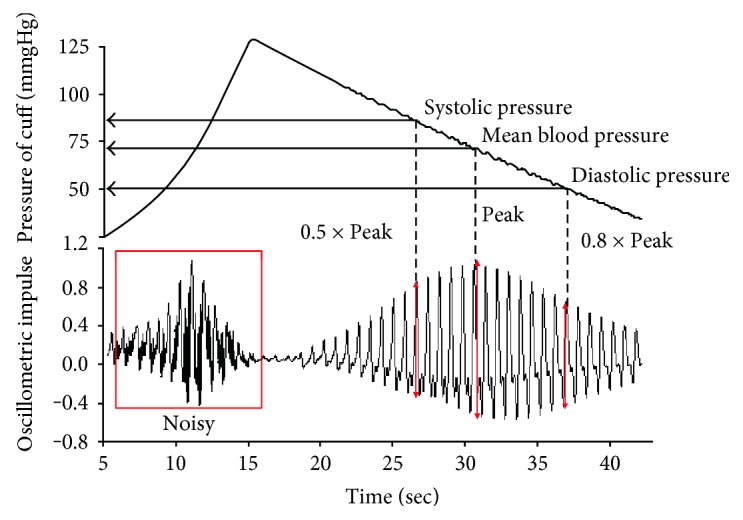
Cuff pressure waveform of oscillometric method.

In general, the SBP and DBP are corresponding to the pressure of 0.5 times and 0.8 times of oscillometric peak, as shown in Figure 1. The estimation of SBP and DBP from oscillometric waveform is important. Lim et al. [17] proposed an improved measurement of blood pressure in terms of extracting the characteristic features from the cuff oscillometric waveform. Their study employed the multiple linear regression (MLR) and the support vector regression (SVR) for the relationship discovery. Moreover, Koohi et al. [18] presented a dynamic threshold algorithm to evaluate trustworthiness of the estimated blood pressure in oscillometry.

However, for conventional oscillometry, a linear active inflating cycle and an inactive deflating cycle were used. Although the inflecting pumping speed is one of the major concerns of a noisy signal, the disturbances and noises are also related to the air turbulences and electrical signal noise generated from pumping motors during the air pumping stage, as shown in Figure 1. With such interference, the determination of BP characteristics would be challenging for conventional oscillometry. In other words, the active inflating cycle contains pumping air turbulence; hence, the active inflating cycle cannot be directly used for precisely determining the BP characteristics. As a consequence, conventional oscillometry measures and determines the BP characteristics at the inactive deflating cycle. For example, the cuff is desired to inflate to a pressure which is in excess of SBP, such as 160 mmHg, and then, it is released below DBP with a linear trajectory in every measurement. The duration of each measurement is taken above 30 seconds. Such a mechanism results in a longer measure time of BP characteristics.

Therefore, this paper proposes a nonlinear trajectory tracking control approach in the inflating and deflating cycles without using conventional pumps. Compared with conventional electronic BP instruments, the novelty of the proposed BP measurement approach is to utilize a variable volume chamber which actively and stably alters the cuff pressure during inflating or deflating cycles. The variable volume chamber is operated with a closed-loop pressure control scheme, and it is activated by controlling the piston position of a single-acting cylinder driven by a screw motor. Therefore, the variable volume chamber could significantly eliminate the air turbulence during the air injection stage when compared to an air pump mechanism.

Furthermore, the proposed active BP measurement approach is capable of measuring BP characteristics during the inflating cycle. Two modes of air injection measurement (AIM) and accurate dual-way measurement (ADM) were proposed, where the AIM mode is desired to significantly reduce the measurement time and the ADM mode is desired not only to reduce the measurement time but also to improve the measurement consistence and precision which can be evaluated in terms of statistical standard deviation. The details are described in the next section.

Finally, this paper is organized as follows.  Section 2 describes the system architecture. Different measurement modes, pressure-trajectory planning, BP measurement algorithm, and active pressure control are introduced in this section. The implementation, including the mechanical design, signal processing, and controller is stated in Section 3. In Section 4, the proposed approach is evaluated with a number of experiments, and a commercial BP instrument is used for comparison; a summary of our study is also conducted. Finally, conclusions and future works are addressed in Section 5.

## 2. System Architecture

**Figure 2:**
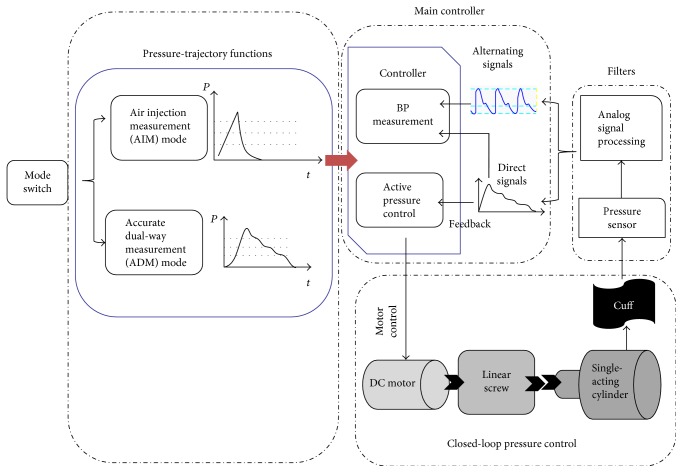
System architecture of the proposed active BP measurement approach.

The system architecture of the proposed active BP measurement approach is shown in Figure 2. It is composed of a single-acting cylinder, a linear screw, a DC motor, and a sphygmomanometer cuff. The stationary single-acting cylinder and the linear screw replace the function of a disturbing air pump which is adopted for most commercial instruments. The single-acting cylinder connects with the cuff through a low elastic rubber tube to form a variable volume chamber. The chamber volume is changed by altering the piston position of a single-acting cylinder which is connected to a linear screw. It is noted that the linear screw is driven by a DC motor. Meanwhile, the changing volume alters the pressure in the cuff. In order to implement a closed-loop pressure control system, a pressure sensor which provides feedback pressure signals is equipped. The signals include alternating signals and direct signals. The controller further deals with the measured signals for BP characteristic recognitions (SBP and DBP) and the closed-loop nonlinear pressure trajectory control in both inflating and deflating cycles. It is noted that the pressure-trajectory functions represent nonlinear pressure trajectory functions for activating the AIM and ADM modes. The details are described in the next subsection.

### 2.1. Introduction of Different Measurement Modes

**Figure 3:**
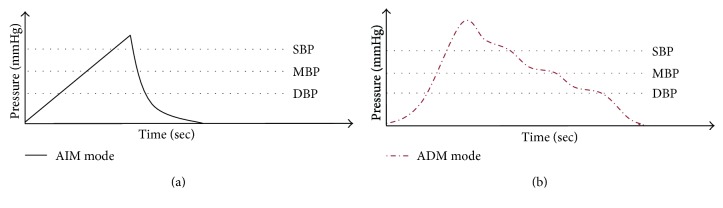
Different measurement modes of this paper: (a) AIM mode; (b) ADM mode.

As described in Introduction, two measurement modes of AIM and ADM were used. The trajectories of pressure for each mode are shown in Figure 3. The measurement modes are switched according to different measurement purposes. The operations of these two modes are described as follows:

1. AIM (air injection measurement) mode: this mode is desired to measure the blood pressure characteristics in the air injection stage. Practically, a linear and stable inflating cycle is generated from a volume-variable single-acting cylinder for BP characteristic measurement; hence, it overcomes the drawback of the conventional oscillometry that the BP characteristics do not determine during inflating cycle. It is noted that the disturbances and noises of conventional BP monitors are related to the air turbulences and electrical signal noise generated from pumping motors during the air pumping stage. With such interference, the determination of BP characteristics would be challenging during air pumping cycle for conventional oscillometry. On the other hand, because of the introduction of stable and stationary air injection of the AIM mode, the cuff pressure is linearly increased. The alternating signal indicated in Figure 2 is processed continuously to detect the DBP and SBP. When the SBP is detected, the air is fast extracted to reduce the measurement time. This mode presents a fast BP measurement solution.
2. ADM (accurate dual-way measurement) mode: the ADM uses a nonlinear inflating and deflating cycles, and the design of nonlinear tracking control in the inflating and deflating cycles aims at reducing the measurement time and the measurement resolution of the oscillometry as well. As shown in Figure 3(b), the ADM contains a fast ramp up inflecting cycle and a variable resolution deflecting cycle. The fast ramp up inflecting cycle determines three rough BP characteristics with a low resolution and fast speed operation. Then, the three rough BP characteristics help the generation of nonlinear deflation cuff pressure trajectory in the continued variable resolution deflecting cycle. As a consequence, more precise BP characteristics can be found due to a higher resolution of slow inflection curve near the region of rough BP characteristics. At the same time, the increasing of trajectory slope during the far rough BP characteristic region is desired to reduce the measurement time.

### 2.2. Pressure-Trajectory Control of ADM BP Measurement

**Figure 4:**
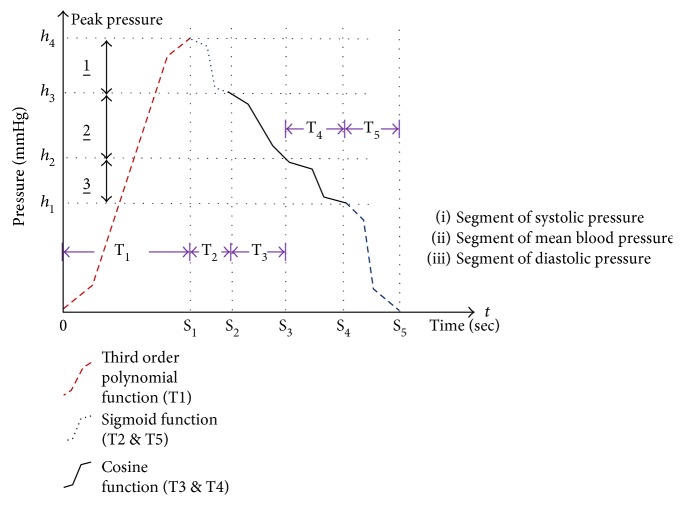
Pressure control curve of BP measurement.

In this subsection, a specific pressure trajectory for achieving the active cuff pressure control of the ADM mode is discussed. Basically, the pressure trajectory is formed with 5-time intervals (i.e., T1 to T5 segments). In order to achieve efficient BP measurements, three different characteristic functions are applied to compose the active pressure trajectory, including sigmoid functions, cosine functions, and third order polynomial functions. As shown in Figure 4, the trajectory of pressure for a complete BP measurement is divided into five stages, that is, from T1 to T5. T1 is the stage of air injection, and T2 to T5 are the stages of air extraction. T1 is used to determine three rough characteristic BP characteristics, including the segment of systolic pressure (SSP), the segment of mean blood pressure (SMP), and the segment of diastolic pressure (SDP). These three rough BP characteristics altered the beginnings and ends of functions of the time segments of T2 to T5. Thus, the trajectories of pressure are automatically adjusted according to each measurement. The decreases of pressures are gentle at the stages which are close to the characteristic points of BP (i.e., roughly detected DBP, MBP, and SBP). Contrarily, the pressure curve segments which are far from characteristic points of BP decrease fast. These five-time segments are categorized as three types of functions, and they are described as follows:

(1) Fast air injection period (T1): In this period, the air is injected to the cuff quickly to roughly obtain the DBP, MBP, and SBP. The pressure curve in this period is desired to be fast increased and smooth. Because the air is injected to the cuff through a DC motor-driven cylinder chamber, a third order polynomial function is suitable for this case. The third order polynomial function provides a gentle slope increasing rate at the beginning and ending of the curve segment so that the DC motor may work smoothly without inducing vibration interference. In addition to the beginning and ending, the curve exhibits fast increasing of the pressure. Because the DBP, MBP, and SBP appeared in the fast increasing pressure duration, the detection of DBP, MBP, and SBP would lose resolution and accuracy. Therefore, the fast air injection period is to roughly obtain the DBP, MBP, and SBP so that a specific air release pressure trajectory (i.e., from T2 to T5) can be desired to improve the accuracy of detecting DBP, MBP, and SBP as well as to reduce the measurement time. Equation (1) shows the third order polynomial function, where *h* is the amplitude; *T* is the period of function; *t* is the time. It is noted that *T* = *S*_1_ and *h* = *h*_4_ in this case.
  (1) $$\begin{array}{c} p\left(t\right)=\frac{-6h}{T^{3}}\left(\frac{t^{3}}{3}-\frac{t^{2}T}{2}\right). \end{array}$$
(2) Non-BP characteristic periods (T2 and T5): In these two periods, there is not any BP characteristic appears. Therefore, the pressure trajectories are designed to be smooth at the beginning and end as well as to be fast at the middle of the period. The design purpose is to reduce the vibration of the motor and to reduce the time. As a consequence, the sigmoid function is applied to the stages of T2 and T5 which are far from characteristic BP points. Therefore, the characteristic of greater slope is suitable for fast air extraction. Equation (2) is the increasing function (*S*_*d*_) and the decreasing function, where *h* is the amplitude; *T* is the period of function; *t* is the time; *t*_*b*_ is the start time of the period. It is noted that *T* = (*S*_2_ − *S*_1_) or (*S*_5_ − *S*_4_); *h* = (*h*_4_ − *h*_3_) or *h*_1_; *t*_*b*_ = *S*_1_ or *S*_4_ in this case.
  (2) $$\begin{array}{c} s_{d}\left(t\right)=\frac{h}{1+e^{2\pi \left(2\left(\left(t-t_{b}\right)/T\right)-1\right)}}. \end{array}$$
(3) BP characteristic periods (T3 and T4): The pressure trajectories in these two periods are important because BP characteristics will be recognized. The trajectory must be smooth and gentle so that the precision of BP characteristic recognition could be improved. The cosine function is applied to the period of T3 and T4 which are close to characteristic BP points. The cosine function has a smaller slope change which is made use of going through the characteristic BP segments. Equation (3) shows the decreasing cosine function, where *h* is the amplitude; *T* is the period of function; *t* is the time. It is noted that *T* = (*S*_3_ − *S*_2_) or (*S*_4_ − *S*_3_); *h* = (*h*_3_ − *h*_2_) or (*h*_2_ − *h*_1_); *t*_*b*_ = *S*_2_ or *S*_3_ in this case.
  (3) $$\begin{array}{c} c_{d}\left(t\right)=\frac{h}{2}\cos\left(\frac{\pi \left(t-t_{b}\right)}{T}\right)+\frac{h}{2}. \end{array}$$

### 2.3. Comparison of Sigmoid and Cosine Functions

**Figure 5:**
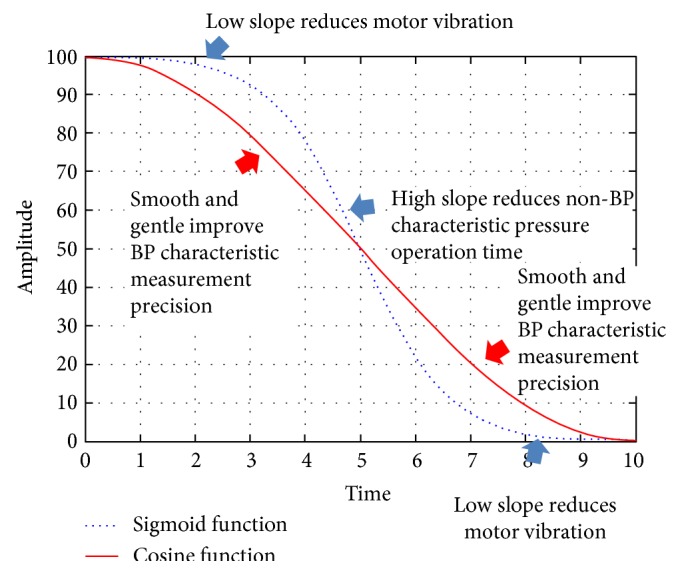
Trajectories of sigmoid function and cosine function.

To illustrate the uses of sigmoid and cosine functions in the aforementioned periods, Figure 5 demonstrates their trajectories. Within specific durations and amplitudes, there are greater changes of slopes in the sigmoid function than in the cosine function in the middles of trajectories. Therefore, sigmoid functions are applied to the noncharacteristic BP segments, and cosine functions are applied to recognize the BP characteristics. In addition, both functions have gentle changes of slopes at the beginnings and the ends. It prevents the DC motor from violent changes of speeds.

### 2.4. Measurement of BP

The proposed BP measurement approach is based on the oscillometric method. When the pressure of cuff is close to MBP, the peak of oscillometric curve occurs. Then, SBP and DBP could be obtained with the corresponding amplitudes of oscillometric pulses. However, there are five modes which are designed in this paper. Except the pressure-increasing measurement mode, other modes obtain BP during the stage of air extraction. Therefore, the algorism of BP measurement must meet the switch of different modes. Thus, the BP measurements are able to obtain in both stages of air injections and air extractions.

The oscillometric pulses are detected from altering signals. The MBP which is the peak of oscillation needs to be recognized first. The SBP and DBP are obtained according to the oscillometric pulses of 0.8 times and 0.5 times of the amplitude of oscillometric peak. Before MBP, the pressure of cuff is increasing that means the air injection is activated, and the BP is obtained during the stage of air injection. Contrarily, before MBP, the pressure of cuff is decreasing that means the air extraction is activated, and the BP is obtained during the stage of air extraction. If the pressure-increasing measurement mode is adopted, the DBP is recognized at the oscillometric pulse of 0.8 times of oscillometric peak. Then, the SBP is recognized at the oscillometric pulse of 0.5 times of oscillometric peak later. If another mode is adopted, the SBP is recognized at the oscillometric pulse of 0.5 times of oscillometric peak. Then, the DBP is recognized at the oscillometric pulse of 0.8 times of oscillometric peak later.

It is noted that the oscillometric peak setting is not time dependent, and it is cuff pressure dependent. Because of using precise closed-loop cuff pressure control scheme, the determination of BP characteristics would be independent of inflation/deflation speed and subject population.

### 2.5. Active Cuff Pressure Control

**Figure 6:**
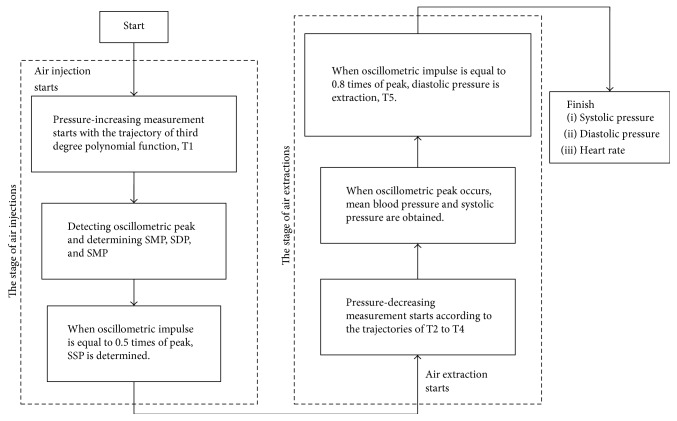
Flow chart of proposed BP measurement.

The proposed active pressure control is implemented based on a PID controller.  Figure 6 shows the flow chart of the proposed BP measurement. The targeted trajectory of pressure which is generated by the system is the input signal, and the pressure sensor which connects with the cuff is the feedback sensor. The PID controller generates PWM signals to the DC motor according to the errors as well as altering the position of piston. At the stage of T1, the system increases the pressure of cuff with a fast speed which is greater than 15 mmHg. In the meanwhile, the oscillometric method is applied to determine the SSP, SMP, and SDP. This procedure determines characteristic functions of extracted stages. The system increases the pressure of cuff until in excess of SBP, and then the system goes to the stage of air extraction. The stage of air extraction is composed of T2 till T5 stages.

At the beginning, because T2 stage is far from the characteristic segment, the sigmoid function is made use of the command signal to decrease the pressure of cuff quickly. On the other hand, T3 and T4 stages include all of the characteristic points. Therefore, the cosine function is made use of controlling the change of pressure. Finally, T5 stage is the end of air extraction, and the sigmoid function is made use of releasing the pressure of cuff. To the pressure-increasing measurement mode, each measurement is finished at the stage of air injection. Therefore, measurement results are obtained at T1 stage and the system fast releases the pressure of cuff that skips the stages of T2 to T5.

## 3. Implementation of Active Blood Pressure Measurement Approach

The pressure of cuff is measured by a pressure sensor. The signals of measured pressure are divided into direct signals and alternating signals. The direct signal which is obtained after a low-pass filter represents the current pressure of cuff. Therefore, the direct signal provides not only BP measurement results but also feedback signals of the controller. The alternating signal is obtained after a band-pass filter. Based on the oscillometric method, oscillometric amplitudes are the important features to detect the SBP and the DBP. When measurements start, the system generates predefined pressure trajectories as the input command. According to the feedback signal of pressure sensor, the controller sends PWM signals to the DC motor and alters the position of single-acting cylinder. The pressure of cuff is changed in terms of altering the volume of chamber. In the meanwhile, the characteristic BP segments are obtained at the air injection stage. The characteristic BP segments adjust the pressure trajectory of air extraction stage to achieve efficient performances.

### 3.1. Design of Proposed BP Measurement Device

**Figure 7:**
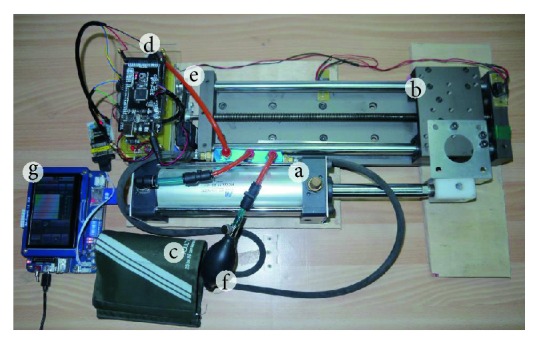
Picture of the proposed BP measurement device, including (a) single-acting cylinder; (b) linear screw; (c) cuff; (d) pressure sensor and analog circuit; (e) DC motor, timing wheel and timing belt; (f) bulb; and (g) monitor.

As shown in Figure 7, a single-acting cylinder with 63 mm in inner diameter and 150 mm in stroke is used. When the piston of the cylinder moves toward the air vent which connects with the cuff, the volume of air in the chamber is compressed. This movement injects the air of chamber into the cuff and increases the pressure. Contrarily, when the piston moves away from the air vent, the air of cuff is extracted and the pressure is decreased.

The single-acting cylinder uses preinjected air in the volume-variable chamber. The closed-loop cuff pressure control is achieved in terms of activating a DC screw motor. The DC screw motor injects the air with relatively stable during the changes of cuff pressure when compared to disturbed air pumping mechanism. In addition, the feedback pressure sensor eliminates the air leaking problems when tracking a specific cuff pressure trajectory. In case of the large amount of air leakage, the screw motor position and cuff pressure feedback can be detected and injected manually with a bulb that was indicated in Figure 7(f).

**Figure 8:**
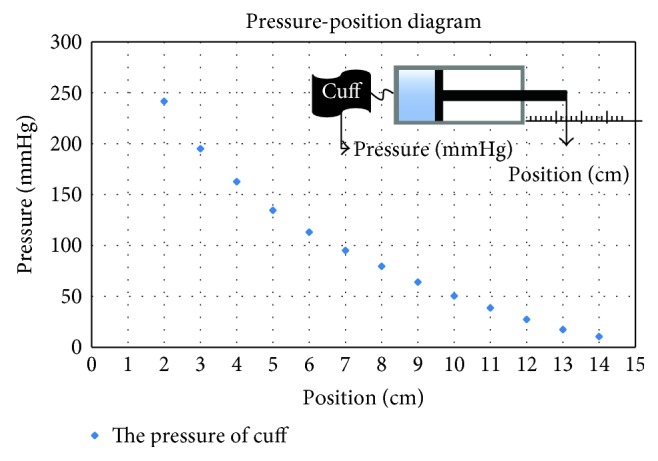
Pressure-position diagram.

The pressure-position diagram of proposed device is shown in Figure 8. In Figure 8, when the position of piston is at 30 mm, the pressure of cuff is about 200 mmHg which is higher than the normal SBP. Therefore, the volume of the selected single-acting cylinder is suitable for this study. In addition, Teflon tapes are affixed to any connections, such as tubes and air vents, in order to prevent the air from leaking. The piston connects with the slide of linear screw. The linear screw is driven by a DC motor. The DC motor with type Faulhaber 2643 series was used, and it is capable of providing 28 mN-m in torque with 24 volts. It transfers the rotary motions to the linear screw through timing wheels and a timing belt. It is noted that the timing wheel and timing belt were formed as a transmission mechanism. The belt and circular wheel were formed with teeth so that they can make sure no slipping effects during mechanical transmission.

### 3.2. Signal Processing of Pressure Signal

**Figure 9:**
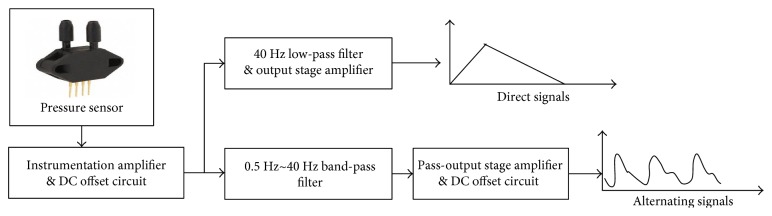
The analog signal processing of measured pressure signals.

**Figure 10:**
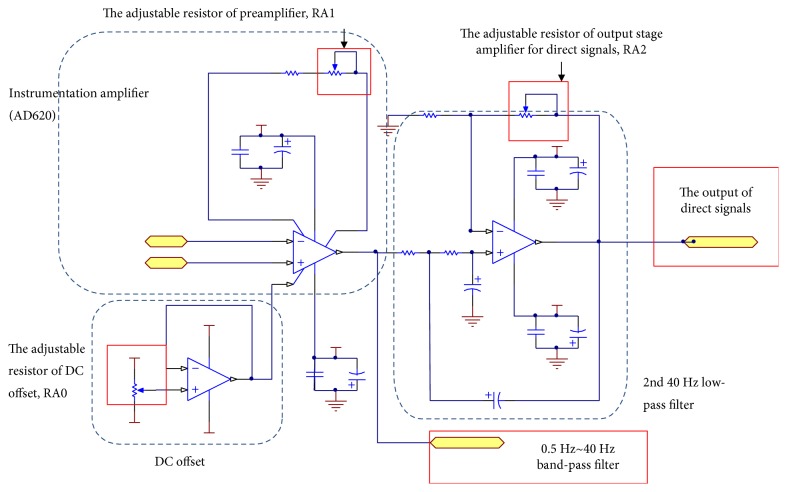
Analog signal processing circuit of direct signals.

**Figure 11:**
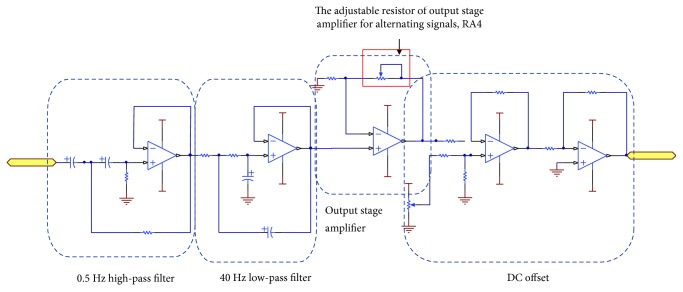
The analog signal processing circuits of alternating signals.

The proposed BP measurement system is equipped with a pressure sensor, Honywell SCC05DN. The sensory range is from 0 mmHg to 250 mmHg, and the output signal is from 25 mV to 26 mV. The measured pressure signals include direct signals and alternating signals. In order to obtain the particular frequencies of signals, an analog signal processing circuit is implemented. The flow chart of analog signal processing is shown in Figure 9. First, the instrumentation amplifier, a preamplifier, is applied to set the gain of signal to 45. There are advantages of high signal to noise ratio and high common-mode rejection ratio. It is noted that the selected instrumentation amplifier outputs the signal from −5 V to 5 V, and the signal is read by an analog to digital converter (ADC) interface. Therefore, a reference terminal potential of the amplifier defines the zero output voltage. The implemented analog circuit is shown in Figure 10. The adjustable resisters of RA0 and RA1 are for adjusting the voltage offset and the gain of preamplifier.  Table 1 lists the resister values and the gains of analog signal processing circuit shown in Figures 10 and 11.

**Figure 12:**
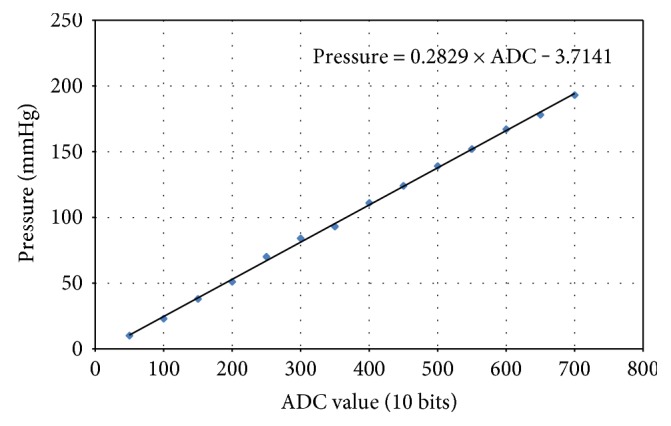
The curve fitting of pressures and ADC values.

In order to obtain direct signals, a 40 Hz 2nd order Sallen-Key low-pass filter is designed to remove the high-frequency noise, as shown in Figure 10. The adjustable resister, RA2, is for adjusting the gain of output stage amplifier. The filtered signal further sets the gain of signal to 2. After detecting the direct signal, the curve fitting approach is applied to find the transformations of pressures and ADC values. A commercial pressure meter is made use of measuring the actual pressure, and the corresponding ADC value is recorded. The curve fitting function is able to be obtained according to experimental results, as shown in Figure 12.

To alternating signals, a band-pass filter with the band from 0.5 Hz to 40 Hz is designed to filter out unwanted signals, as shown in Figure 11. The normal heart beat ranges from 1 Hz to 1.7 Hz (60 bpm to 100 bpm), and the frequency of environment noises is 60 Hz. Therefore, the band-pass filter is composed of a 0.5 Hz 2nd order Sallen-Key high-pass filter and a 40 Hz 2nd order Sallen-Key low-pass filter. The arterial signals are obtained by the band-pass filter. The amplitudes of output signals are approximately from 1 mV to 20 mV. Therefore, an output stage amplifier sets the gain of signal to 90, and the output signal ranges from 0 volts to 5 volts.

### 3.3. Controller of Active BP Measurement

An 8-bit AVR microcontroller is employed as a main controller. There are 15-channel 10-bit A/D converters and 10-channel 1 MHz PWM output interfaces. The main controller deals with the measured pressure signals which were processed after the analog circuits. The signals including direct signals and alternating signals are further used as the feedback signals and oscillometric signals for proposing the active pressure control and the deriving algorism for BP measurement. The controller generates PWM (pulse width modulation) signals to control the speed of DC motor and to alter the position of piston. Then, the cuff pressure is adjusted according to measured pressures and desired pressures. It is noted that the mentioned BP measurement modes and algorisms are implemented in a main controller to achieve the purpose of a real-time control.

### 3.4. Design of DC Motor Driver Module

**Figure 13:**
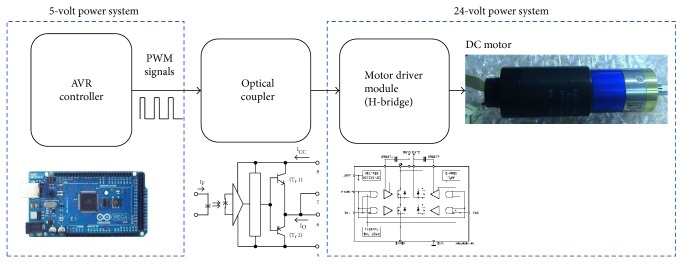
Architecture of DC motor driver module.

The main control generates PWM signals to control the DC motor. However, the voltage levels of PWM signals which are generated by the main controller are 5 volts. In addition, the insufficient currents cannot actuate the DC motor. Therefore, a DC motor driver with type L6201 is introduced to transform the voltage level to 24 volts. The maximum current output of the motor driver is 1 A, and the operating frequency reaches 100 kHz. Therefore, the specification of the selected motor driver meets the required operating frequency of DC motor, 20 kHz. Because the electromagnetic interference (EMI) which is caused by the motor affects the performance of circuit, an optical coupler is employed to prevent the main controller from disturbance, as shown in Figure 13.

## 4. Experiment and Results

Several experiments are arranged to evaluate the performance of the proposed active BP measurement approach. First, the system responses of different parameters in the PID controller algorithm are observed. Second, the control of desired pressure trajectory is verified to evaluate the performance of the main control. Third, different measurement modes are executed to verify the BP measurement algorism. Finally, five subjects are employed to evaluate the proposed BP measurement approach, and a commercial electronic BP measurement instrument is further evaluated for comparison.

### 4.1. Experiments of Active Cuff Pressure Control

**Figure 14:**
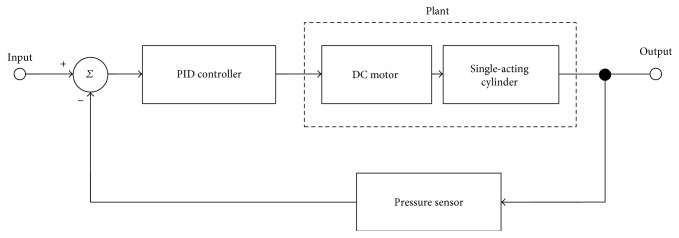
The block diagram of the active pressure control.

**Figure 15:**
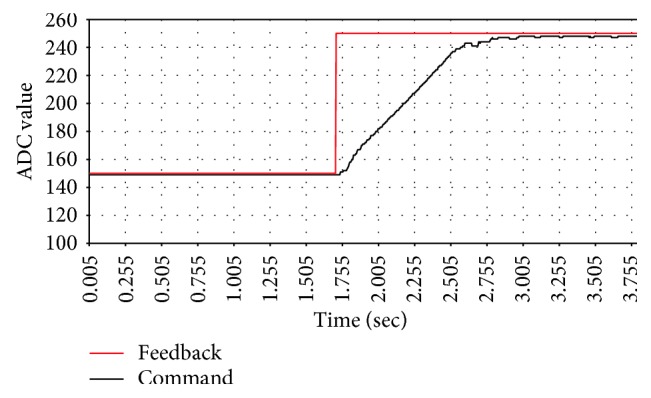
The step response of *P* = 30, *I* = 0.0015, and *D* = 8.

**Figure 16:**
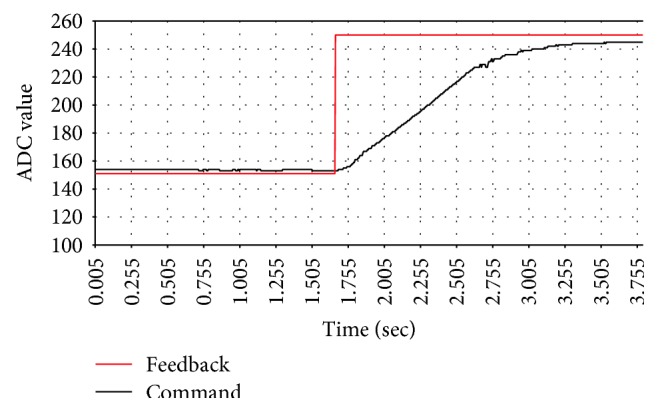
The step response of *P* = 60, *I* = 0.0015, and *D* = 8.

**Figure 17:**
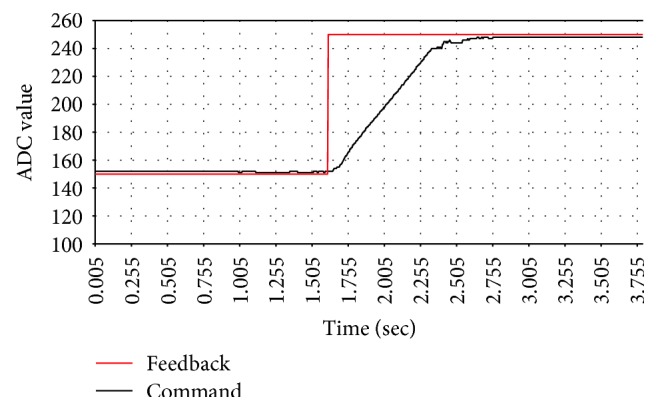
The step response of *P* = 60, *I* = 0.0030, and *D* = 8.

The block diagram of the active pressure control is shown in Figure 14. In order to evaluate the performance of the controller, the step response with different control parameters is observed. Each step response diagram is shown from Figures 15, 16, and 17.

Table 2 lists the step response of each control parameter. From the experimental result, *P* is increased to shorten the response time and *I* is adjusted to eliminate the steady state error. The PID parameters determine the performance of pressure control and directly affect measurement results. From Table 2, the PID controller with parameters of *P* = 60, *I* = 0.003, and *D* = 8 has shorter response time and smaller steady state error than the other parameters. These parameters are selected for the following experiments.

### 4.2. Verification of Active Cuff Pressure Control

**Figure 18:**
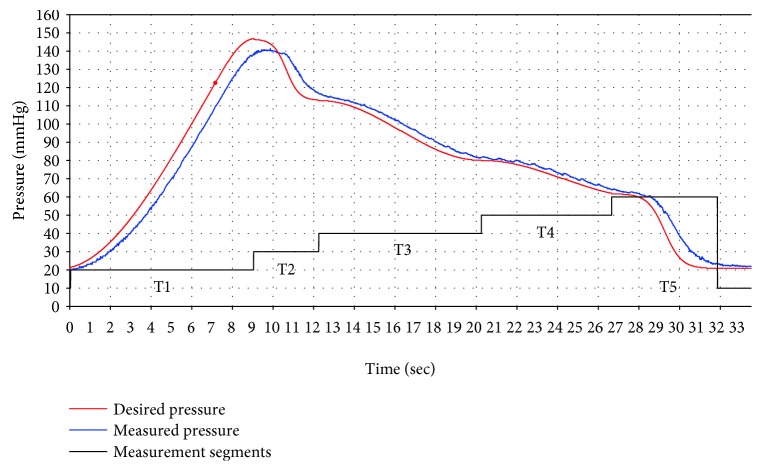
The trajectories of desired pressure and measured pressure (ADM mode).

The proposed BP measurement approach includes AIM and ADM measurement modes. In this experiment, the ADM mode is selected to verify the performance of PID controller with respect to response time and pressure feedback. The ADM mode includes all of the required trajectories of pressure waveforms from T1 to T5 in a measurement. Therefore, the result covers all of the verifications of five measurement modes. As shown in Figure 18, the blue curve is the desired pressure and the red curve is the measured pressure. The controlled functions are changed according to different measurement stages, as being plotted with the black line. Because of the response time, the desired pressure and the measured pressure are not equal. The response time was approximately from 0 sec to 1 sec, and the errors ranged from 5 mmHg to 10 mmHg.

### 4.3. Verification of BP Measurement

**Figure 19:**
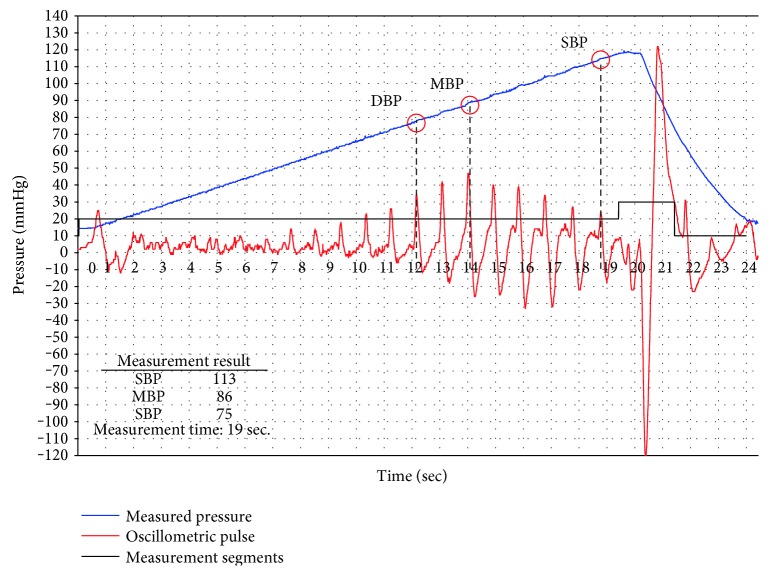
The measurement result of the AIM mode.

**Figure 20:**
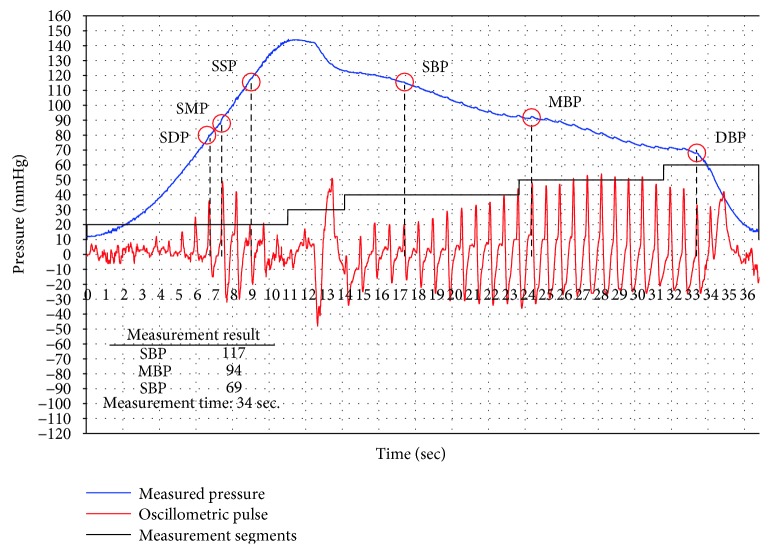
The measurement result of ADM mode.

Based on the above ADM mode experiment and verification, the proposed measurement modes of AIM and ADM are further examined by integrating BP measurement algorisms. The AIM mode experiment is shown in Figure 19. The BP measurement result is obtained at the stage of air injection, and then the cuff is deflated. The blue curve indicates the measured pressure; the red curve is the oscillometric impulse; the recognized BP characteristics are indicated with red circles with SBP, MBP, and DBP symbols. For a complete measurement of AIM mode experiment setting in Figure 19, it took less than 20 s to obtain SBP. The ADM mode experiment is shown in Figure 20. In this experiment, it took 34 s to finish a complete ADM mode measurement. However, to improve the AIM mode accuracy, the linear trajectory could be desired as a slower pressure increasing curve. Similarly, to reduce the ADM mode measurement time, the nonlinear trajectory could be also desired. The new setting will be used for comparison with a commercial BP monitor.

### 4.4. In the Comparison with Commercial Electronic BP Device

In order to verify the feasibility and performance of proposed AIM and ADM modes, a commercial electronic BP instrument (with type: Microlife BP A200) was employed for comparisons.

**Figure 21:**
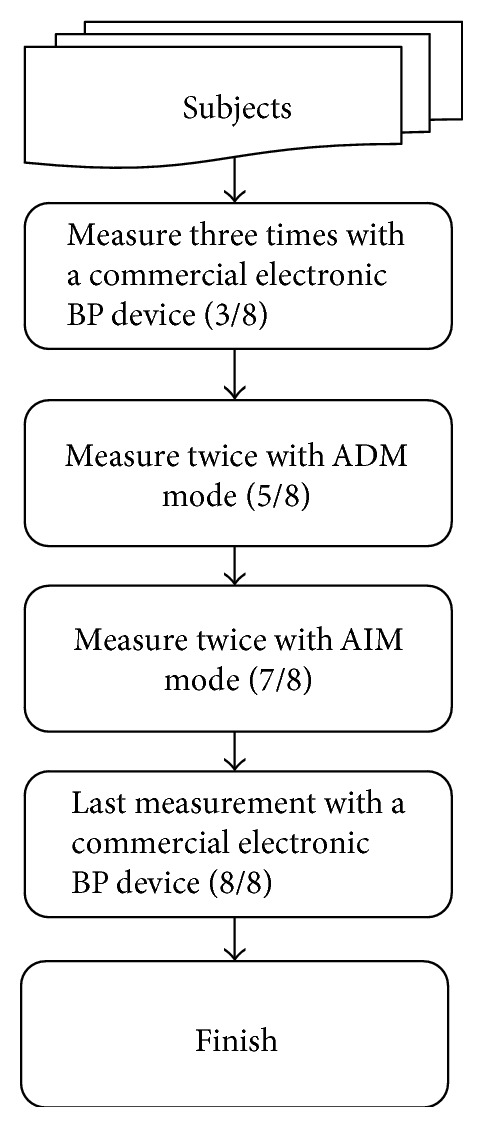
The flow chart of BP measurement experiment.

Five healthy subjects were asked to proceed continuously with 8-time BP measurements. SBP, DBP, and measurement time were recorded in each experiment. There is a one-minute interval between two measurements to avoid the problem of losing arterial compliance. The arrangement of the experiment is designed as Figure 21. At the beginning, each subject is measured by a commercial electronic BP device for three times. Then, the proposed ADM mode is executed for the next two measurements. The AIM mode provides the 6th and 7th experiments. Finally, the commercial electronic BP instrument provided the last one measurement.

Table 3 summarized the measurement results of the abovementioned experiments. Compared with the commercial instrument, there was a difference of 5 mmHg in average between the commercial device and the proposed ADM mode and a difference of 15 mmHg in average between the commercial device and the AIM mode. In addition, the average measurement time significantly decreased. The commercial device is about 38 seconds; the ADM mode is about 32 seconds; and the AIM mode is 25 seconds. According to the above healthy subject experiment results, the AIM reduced 34.21% and the ADM reduced 15.78% of the measurement time when compared to a commercial BP monitor.

On the other hand, the standard deviation of measurements of the same healthy subject at the same time was also evaluated to examine the performance of precision. With this experiment setting, a young healthy subject, 24 years old, was investigated. The same commercial BP monitor was used for the comparisons with AIM and ADM modes. Three different methods were examined for 11 times to obtain statistics performance. The results are shown in Table 4. It is noted that the time courses of the pressure trajectory were the same as the experiments shown in Table 3. Apparently, the ADM performed much consistently (i.e., less standard deviation) in the measurements when compared to a commercial BP monitor. The AIM performed a little worse performance in the standard deviation when compared to a commercial BP monitor.

It is noted that this experiment setting is not for the BP accuracy comparison, because our study was based on home-made circuits and machines. The circuit and mechanical drive designs were not certificated. Hence, bias and interference would be a concern. Hence, the statistical standard deviation evaluation would be a method to examine the benefits from nonlinear variable pressure control trajectory for improving the resolution within the near BP characteristic regions.

In summary, according to the experiment results, the benefits of the proposed active variable volume chamber method are the following:

1. The active variable volume chamber method could produce stationary injection air so that it can obtain the BP characteristics during injecting stage to reduce the measurement time.
2. The active variable volume chamber method may design air injecting and releasing pressure curves to discover the characteristics of BP curve in a time-efficient manner.
3. Such an approach could be used for the development of automated blood pressure measurements [19, 20].
4. Medical mechatronics integration would bring novel healthcare application which considers the techniques of the biomedical signal processing of cuff pressure sensors as well as the control of motor-activated variable volume cuff chamber [21].

## 5. Conclusions and Future Works

In this paper, a BP measurement approach based on an active control scheme of cuff pressure is proposed. It automatically adjusted the trajectories of pressure to achieve a highly efficient measurement performance. Compared with conventional BP instruments, the dedicated processes of cuff inflation and deflation resulted in a longer measurement time. An actuated single-acting cylinder provides the targeted pressure of cuff by altering the position of piston. It significantly eliminated the air disturbances during air pump. According to the characteristic BP segments, the functions of trajectories are automatically generated to achieve the considerations of both measuring time and resolution. The single-acting cylinder is actuated by a DC motor and a linear screw. The active pressure control is implemented in terms of a closed-loop pressure control scheme. The experiments verified the system performance. According to the healthy subject experiment results, the AIM reduced 34.21% and the ADM reduced 15.78% of measurement time when compared to a commercial BP monitor. The standard deviation of measurements of the same healthy subject at the same time was also evaluated to examine the performance of precision. The ADM performed much consistently (i.e., less standard deviation) in the measurements when compared to a commercial BP monitor.

In the future, the control parameters of system are optimized with respect to characteristic models, such as the systems of single-acting cylinder and cuff. Thus, the stability and efficiency of control system could be further improved. Meanwhile, the real patient with an IRB application will be done to examine the clinical feasibility. Besides, processing the verification with IEC EN 1060-4 standard will be the next plan of this work.

## Figures and Tables

**Table 1:** The resistor values of implemented analog circuit.

Number	Resistor value	Function	Gain
RA1	10 k*Ω*	Adjusting the gain of preamplifier	45
RA2	1.86 k*Ω*	Adjusting the gain of direct signal of output stage amplifier	1.86
RA4	90.8 k*Ω*	Adjusting the gain of alternating signal of output stage amplifier	90

**Table 2:** The step responses of different PID parameters.

	*P* = 30, *I* = 0.0015, *D* = 8 (Figure 14)	*P* = 60, *I* = 0.0015, *D* = 8 (Figure 15)	*P* = 60, *I* = 0.0030, *D* = 8 (Figure 16)
Response time (sec)	1.7	1.0	1.0
Steady state error (ADC)	10.0	5.0	3.0

**Table 3:** Measurement results of proposed and commercial BP device of five healthy subjects (unit: mmHg).

SBP/DBP mmHg	Subject 1	Subject 2	Subject 3	Subject 4	Subject 5	Average measurement time
Method						
Commercial electronic BP device (1st measurement)	115/77	132/61	113/74	103/53	107/78	**38**
Commercial electronic BP device (2nd measurement)	117/77	133/53	107/64	89/51	99/63	
Commercial electronic BP device (3rd measurement)	116/74	137/60	100/62	87/48	101/64	
Commercial electronic BP device (8th measurement)	121/82	123/65	105/59	87/52	105/67	
Average	**116/77**	**131/59**	**106/64**	**91/51**	**103/68**	
ADM mode (4th measurement)	114/75	126/70	114/66	91/64	107/60	**32**
ADM mode (5th measurement)	119/80	125/71	109/67	84/47	110/71	
Average	**116/77**	**125/70**	**111/66**	**87/55**	**108/65**	
AIM mode (6th measurement)	131/66	125/65	129/80	94/52	123/68	**25**
AIM mode (7th measurement)	121/75	139/70	114/65	86/53	112/65	
Average	**126/70**	**132/66**	**121/72**	**90/52**	**117/66**	

**Table 4:** Statistics results of proposed and commercial BP device of a healthy subject with 11 trials (unit: mmHg).

	Mean SBP	SBP STDEV	Mean DBP	DBP STDEV
Microlife	107.36	4.17	70.45	3.64
ADM mode	108.54	3.72	68.36	3.25
AIM mode	112.72	4.22	67.27	3.82
